# Role of carboxylic organic molecules in interfibrillar collagen mineralization

**DOI:** 10.3389/fbioe.2023.1150037

**Published:** 2023-04-05

**Authors:** Abhishek Indurkar, Rajan Choudhary, Kristaps Rubenis, Janis Locs

**Affiliations:** ^1^ Rudolfs Cimdins Riga Biomaterials Innovations and Development Centre of RTU, Institute of General Chemical Engineering, Faculty of Materials Science and Applied Chemistry, Riga Technical University, Riga, Latvia; ^2^ Baltic Biomaterials Centre of Excellence, Headquarters at Riga Technical University, Riga, Latvia

**Keywords:** carboxylate organic compound, calcium phosphate, bone, interfibrillar collagen, mineralization

## Abstract

Bone is a composite material made up of inorganic and organic counterparts. Most of the inorganic counterpart accounts for calcium phosphate (CaP) whereas the major organic part is composed of collagen. The interfibrillar mineralization of collagen is an important step in the biomineralization of bone and tooth. Studies have shown that synthetic CaP undergoes auto-transformation to apatite nanocrystals before entering the gap zone of collagen. Also, the synthetic amorphous calcium phosphate/collagen combination alone is not capable of initiating apatite nucleation rapidly. Therefore, it was understood that there is the presence of a nucleation catalyst obstructing the auto-transformation of CaP before entering the collagen gap zone and initiating rapid nucleation after entering the collagen gap zone. Therefore, studies were focused on finding the nucleation catalyst responsible for the regulation of interfibrillar collagen mineralization. Organic macromolecules and low-molecular-weight carboxylic compounds are predominantly present in the bone and tooth. These organic compounds can interact with both apatite and collagen. Adsorption of the organic compounds on the apatite nanocrystal governs the nucleation, crystal growth, lattice orientation, particle size, and distribution. Additionally, they prevent the auto-transformation of CaP into apatite before entering the interfibrillar compartment of the collagen fibril. Therefore, many carboxylic organic compounds have been utilized in developing CaP. In this review, we have covered different carboxylate organic compounds governing collagen interfibrillar mineralization.

## Introduction

**FIGURE 1:**
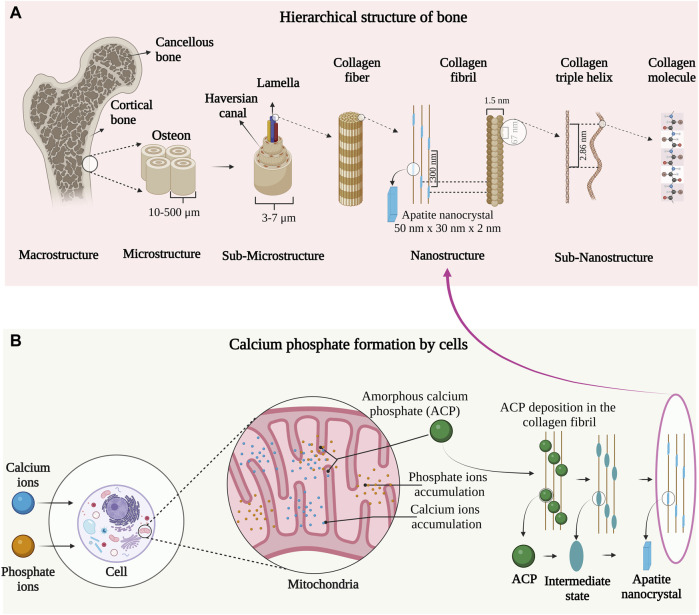
**(A)** Hierarchical organization of bone from macro to the nanoscale. Image inspired by the previous publication of (Rho et al., 1998). **(B)** Mitochondrial-dependent cellular mechanism of apatite nanocrystal formation. The image is inspired by the fifth jubilee lecture on mitochondria and calcium ion transport (Lehninger, 1970).

Bone is a heterogenous and anisotropic nanocomposite. The components are arranged hierarchically into several structural levels shown in Figure 1A. The macro-structure consists of cancellous and cortical bone which is divided into microstructure composed of the Haversian system and osteons. The microstructure is divided into sub-microstructures consisting of lamella that are further divided into fibrillar collagen and apatite nanocrystal nanostructure (Fratzl and Weinkamer, 2007). The final sub-nanostructure consists of elements such as minerals, collagen, non-collagenous proteins, and small organic moieties (Heikkilä, 2011). The remarkable mechanical and remodeling capabilities of bone arise from the nanostructure level of collagen fibril and apatite nanocrystal (Liu et al., 2016).

The mineral component of bone is formed by a mitochondrial-dependent cellular process as indicated in Figure 1B. In the late ’90s, Albert L. Leininger and coworkers first observed amorphous calcium phosphate (ACP) synthesis in the mitochondria of cells. According to their observations, Ca^2+^ and phosphate ions in blood plasma are transported into the cell cytoplasm and pumped into the inner wall of mitochondria on a specific carrier by energy-yielding electron transport (Rossi et al., 1967). The ions are accumulated in the mitochondria and once the solubility (concerning ACP) is exceeded, the precipitation occurs in the form of ACP micro-packets. These micro-packets are believed to be stabilized in the mitochondria by an organic moiety termed the “Howard factor.”

In collagen, the charged amino acids are present in both the 67 nm overlap and 40 nm gap zone of collagen providing nucleation sites that regulate the conversion of ACP into an organized array of apatite nanocrystals (Nudelman et al., 2010). It is believed that the net positive charge on the C-terminal of the collagen molecule allows ACP to settle in the collagen fibril. However, auto-transformation of ACP into apatite nanocrystal before entering the interfibrillar compartment of the collagen fibril was observed. Studies have shown that organic carboxylic compounds regulate the conversion of ACP to apatite by hindering auto-transformation. Therefore, many carboxylic compounds have been utilized in developing calcium phosphates carboxylate (Xie and Nancollas, 2010; Yokoi et al., 2022).

Despite these discoveries, the complex process of collagen interfibrillar mineralization is not known completely understood. In the first section of this review, we have focused on the structural and chemical aspects of collagen. In the second half of this review, we have focused on the postulated mechanisms and role of the different carboxylic organic compounds responsible for interfibrillar collagen mineralization.

### Structural and chemical properties of collagen

**FIGURE 2:**
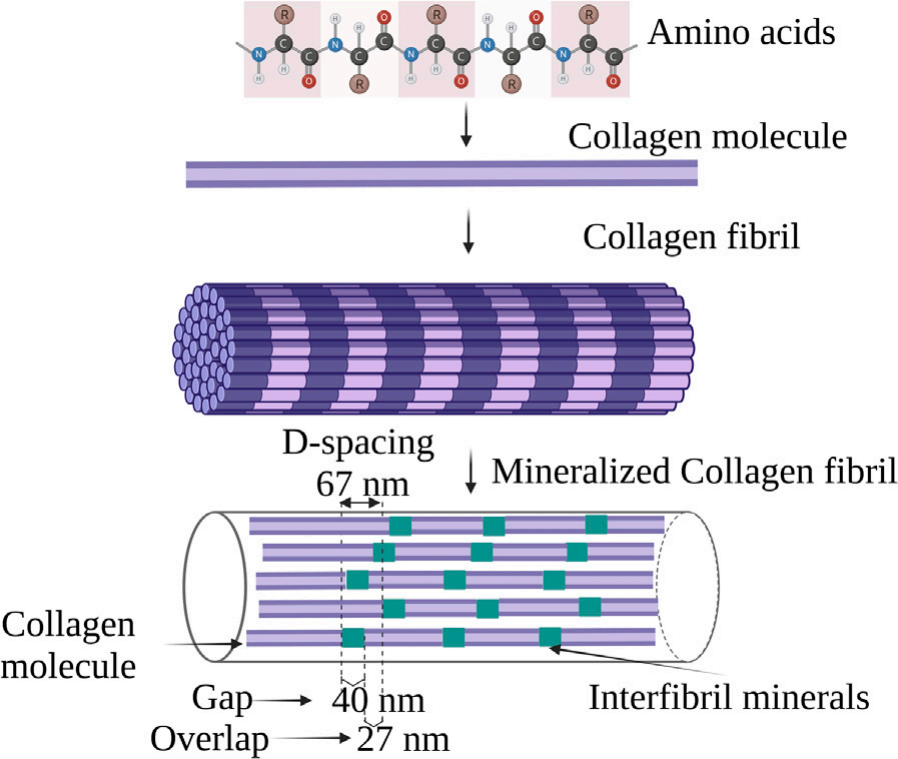
Schematic illustration of the hierarchical structure of collagen.

Collagen type I accounts for 90% of the total organic component of the bone (Fuchs et al., 2009). Structurally collagen consists of a subunit known as tropocollagen which is 300 nm long and 1.5 nm wide “rod” consisting of three polypeptide strands. Each polypeptide strand is a left-handed helix, and all three polypeptide strands are twisted to form a right-handed triple helix (Quan and Sone, 2013) as shown in Figure 2. The collagen fiber bends in a tube-like pattern indicating that the composition is not homogenous laterally, but consists of a hard shell and soft core (Gutsmann et al., 2003). In collagen, 64% of non-polar amino acids comprise glycine, proline, and alanine whereas the hydroxyl residue accounts for 16% and majorly consists of hydroxyproline and serine. The acidic, basic, and amide side chains account for the remaining 20% of the collagen structure. Collagen possesses two distinct hydration phenomena depending upon the pH 1) Swelling induced in the neutral salt solution, described as lyophilic or Hofmeister effect; 2) Swelling in acidic or basic solutions, described as the Donnan effect (Bear, 1952).

In bone, the collagen triple helix is arranged in a parallel staggered array in which collagen molecules are shifted by a distance D concerning their adjacent collagen molecule, after their self-assembly into collagen fibrils as indicated in Figure 2 (Glimcher et al., 1981). One D-repeat consists of a complete collagen sequence of 67 nm and the distance between two tropocollagen subunits measures 40 nm termed a gap zone (Landis et al., 2006). The nucleation site of apatite nanocrystal lies in the gap zone of collagen where it achieves the c-axis orientation parallel to the long axis of collagen (Parvizi and Kim, 2010). The intermediate state of CaP such as ACP and/or octa-calcium phosphates (OCP) (Lehninger, 1970; Brown et al., 1987) are presumed to be nucleated in the gap zone of collagen that results in apatite nanocrystal formation.

The remarkable mechanical property of bone arises from the complex association of the apatite nanocrystal and collagen nanocomposite. The initial studies of interfibrillar collagen mineralization were conducted by transmission electron microscopy (TEM) (Weiner and Traub, 1986) followed by electron tomography (ET) analysis (Landis et al., 1993; Landis et al., 1996). These studies revealed that the orientation of apatite nanocrystals may be regulated by confinement in the collagen fibrils forming a deck of card conformation. The cylindrical nanopores in the collagen can direct the oriented growth of apatite nanocrystals *in vivo* (Olszta et al., 2007). However, this suggestion cannot be considered without detailed ultrastructure of the collagen gap zone. Recently, the detailed model of interfibrillar collagen mineralization was presented by Xu and coworkers. The analysis was performed on the surgical waste material from the tibia fracture of a ten-year-old healthy female. X-ray diffraction (XRD), Electron microscopy and tomography analysis have revealed, the dimension (∼65 nm × 20 nm × 3 nm) of the interfibrillar crystals that possess curved or propeller-like morphology. Extracellular apatite nanocrystals of similar dimensions were also observed which may potentially proliferate into the fibers. The charged amino acids in the collagen are considered to regulate and induce the nucleation of apatite nanocrystals. The study delivered three aspects a) Interfibrillar apatite nanocrystal orientation, b) structure of collagen gap zone, and c) apatite-collagen interaction (Xu et al., 2020).

### Nucleation site in collagen

The ultrasound studies revealed that the apatite nanocrystal deposition in extracellular space and arrangement on collagen fibers was not random but aligned. The apatite nanocrystal was deposited specifically along the axial region of collagen fibers. Electron micrographs showed physiochemical interactions, organizing CaP in 700 Å axial repeat of the collagen fibers. Moreover, the c-axis of apatite nanocrystals is aligned to the long axis of the collagen which may be responsible for the nucleation of apatite nanocrystals in c-axis orientation (Landis, 1986). This eliminates the possibility of orientation through mechanical pressure generated by the dense packing of many apatite nanocrystal crystals.

The charged amino acids such as glutamic acid, hydroxyl-serine, aspartic acid, lysine, arginine, and histidine were found in several two-dimension structure of type-I collagen molecular segment as well as in the adjacent segments of the triple helix chain. The charge distribution of the peptide in the collagen chain offers charged domain that serves as center for apatite formation (Landis and Silver, 2009). The locations containing these specific amino acids have stereochemical configurations that provided binding sites for calcium and phosphate ions. Moreover, the ions are sufficiently close to stimulating ion interaction and formation of apatite nucleation sites (Höhling et al., 1974). In the quasi-hexagonal model, the lysine (at residue 108) and the glutamic acid (at residue 110) in a segment I and the glutamic acid (at residue 815) and arginine (at residue 816) in segment 4 result in a pocket that can accommodate calcium and phosphate ions. The ion binding efficiency also increases in the same gap zone through interaction with glutamic acid (at residue 116) in segment 1, arginine (at residue 350) in segment 2, arginine/aspartic acid (at residue 581 and 582, respectively) and glutamic acid/arginine (at residue 815 and 816, respectively) in segment 4 (Hulmes and Miller, 1979). The stereochemical configuration of the amino acids present within or between the collagen segments provided calcium and phosphate binding sites that aid in the formation of nucleation centers. Recently Silver and Landis (2011) provided more consideration in collagen mineralization; a) orientation of charged side chain, b) collagen crosslinking, and c) impact of non-collagenous molecules.

**FIGURE 3:**
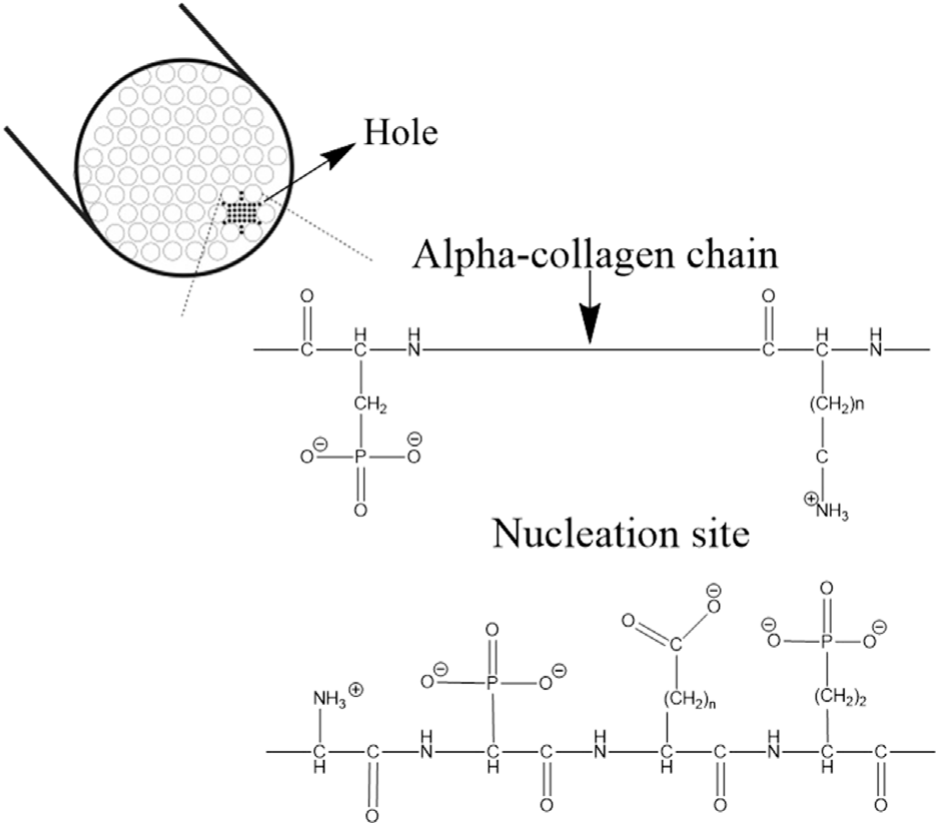
Nucleation sites in the hole zone of the alpha-collagen fibril. The image is inspired by the work of (Glimcher, 1984).

Experimental studies have shown that the synthetic ACP/collagen combination alone is not capable of initiating the nucleation of apatite rapidly (Bradt et al., 1999). Therefore, it was understood that there is the presence of a nucleation catalyst governing the process (Wang and Nancollas, 2008). The nucleation catalyst was defined as an organized organic molecule, containing specific reactive side chains arranged in specific stereochemical and electrochemical arrays establishing a nucleation site as shown in Figure 3. This allows precise cellular control and molecular localization of the interfibrillar collagen mineralization process (Bonar and Glimcher, 1970; Glimcher, 1979).

### ACP and role of Howard factor

Studies of infant rib bone revealed the presence of a non-crystalline phase before the initiation of the “interfibrillar collagen mineralization front” (Robinson and Watson, 1952). Following, in 1956, Jackson and Randall (2008) studied the periosteal bone growth in fowl embryos and discovered an electron-opaque substance in a newly formed fibrous matrix. This opaque substance was considered an amorphous phase of mineral. Further, in 1959, a similar finding was reported while investigating parietal bone in mice. They the provided first morphological evidence of the ACP (Molnar, 1959).

Finally, in 1966, the first experimental studies on ACP were demonstrated (Harper and Posner, 2016). They observed that the non-crystalline counterpart was evenly distributed in osseous tissue in several mammalian species. Further, they described that 40% of the bone mineral in the femur of adult humans, cows, and rats was comprised of the non-crystalline phase (Termine and Posner, 1966). Additionally, Posner (1969) confirmed bone mineral as poorly crystalline calcium phosphate (PCCP) which was not identical to the structure and composition of hydroxyapatite (HaP). They proved that bone consists of physically and chemically divergent phases of CaP. Further studies concluded that the bone consists of a large amount of non-crystalline or ACP apart from apatite nanocrystals.

Along with ACP, parallel studies were undergoing to understand the role of cells in the formation of CaP. In the fifth Jubilee lecture of Lehninger (1970), the role of mitochondria and the active Ca^2+^ ion transport was demonstrated in detail. The major topics such as Ca^2+^ ion a) specificity, b) tissue distribution, c) transport mechanism, d) affinity binding, e) relaxation cycle in muscle, f) formation of calcium phosphate granules, and g) role of mitochondria in interfibrillar collagen mineralization was thoroughly discussed. The key findings are highlighted in the following paragraph.

The rat kidney mitochondria can accumulate enormous quantities of Ca^2+^ depending on the presence of respiratory substances such as isocitrate, succinate, adenosine triphosphate (ATP), Mg^2+^, and/or inorganic orthophosphate (Vasington and Murphy, 1962). In the following year, the relationship between the number of electrons flowing through the respiratory chain and the number of Ca^2+^ ion accumulation was identified. The stoichiometric uptake of both Ca^2+^ and phosphate ions was synergistic with electron transport in such a way that 1.7–2.0 Ca^2+^ ions and 1.0 phosphate ions accumulate per pair of electrons sloping each of three energy-conserving sites of the respiratory chain (Bielawski and Lehninger, 1966). Furthermore, it was also observed that the Ca^2+^ arouses respiration of mitochondria in such a way that two Ca^2+^ ions capitulate the same amount of oxygen as required by one Adenosine diphosphate (ADP) (Carafoli and Lehninger, 1964). The accumulation of Ca^2+^ ions is completed by the ejection of H^+^ ions (Rossi et al., 1966). This is one of the major findings of the relationship between Ca^2+^ and anion accumulation.

Mitochondria are accumulated with Ca^2+^ and diverse types of anions under different conditions. Thus, Ca^2+^ ions are bound to the inner membrane of mitochondria until a perfect anion is available and in the presence of the permanent anion, Ca^2+^ appears in the matrix. ATP or ADP is required in the formation of ACP that accumulates in the mitochondria. This leads to massive loading of mitochondria with ACP. However, the role of ATP in the process remains unknown (Lehninger, 1970).

**FIGURE 4:**
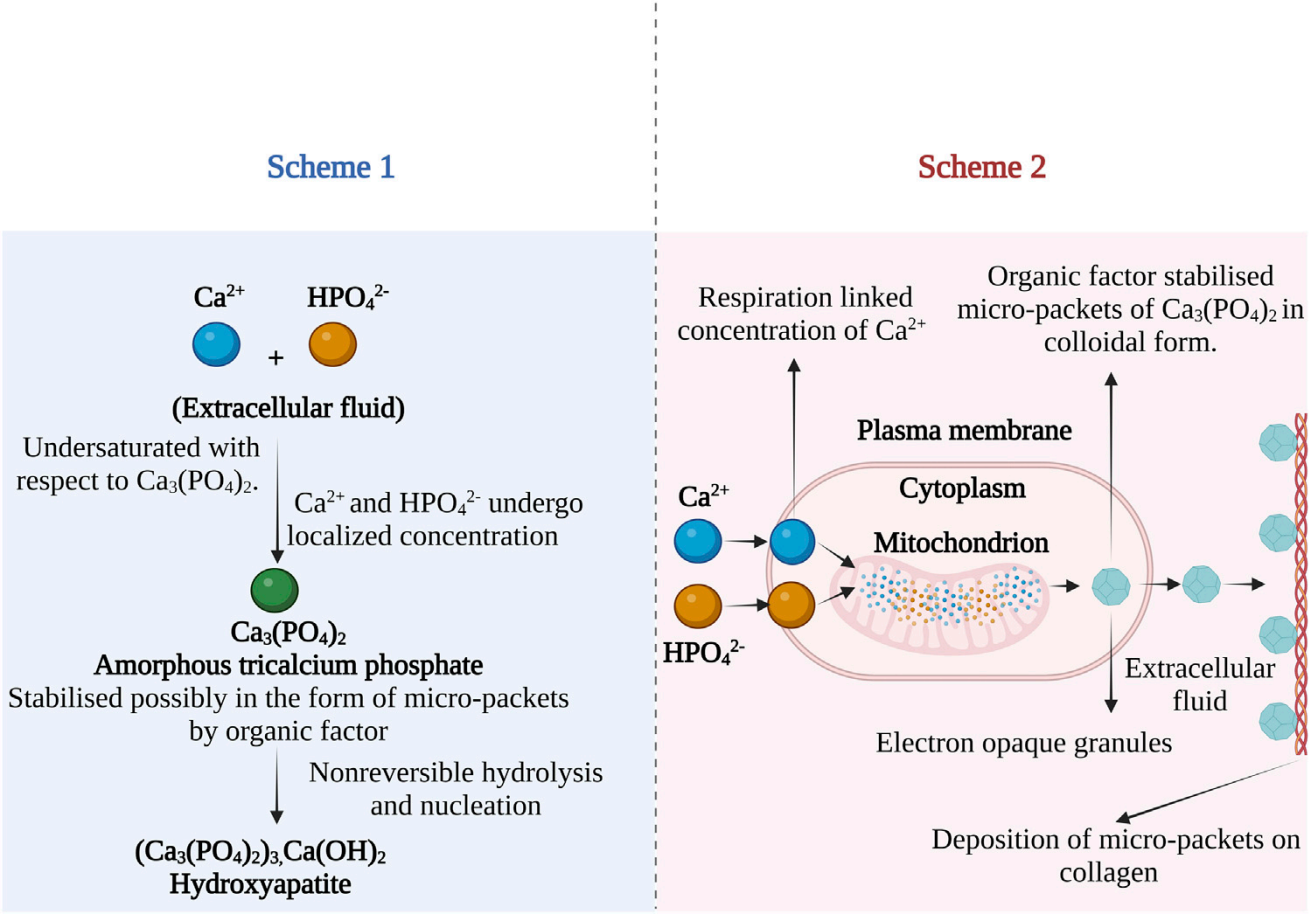
Possible mechanism of bone apatite formation from ACP in the presence of biological inhibitor. Scheme 1—apatite formation mechanism in extracellular fluid and Scheme 2—mitochondrial-dependent cellular mechanism of bone apatite formation. The images are inspired by the fifth jubilee lecture on mitochondria and calcium ion transport of (Lehninger, 1970).

Based on the experimental evidence of cellular machinery involved in CaP formation, Leininger and co-workers presented two theories on the formation of ACP and its conversion to apatite. According to the first theory, the formation of bone apatite in the presence of a biological inhibitor was presented as shown in Figure 4 (Scheme 1). The process is initiated in interstitial fluid, where the Ca^2+^ and phosphate ions in the presence of biological factor (presumed to be Howard factor) undergo localized concentration to exceed the solubility of ACP. The role of the Howard factor was crucial as it prevents the non-biological formation of apatite from free Ca^2+^ and phosphate ions. Further, the ACP micro-packets formed in association with the Howard factor undergo non-reversible hydrolysis followed by nucleation and are later converted to the apatite nanocrystal.

The Second hypothesis describes the mitochondrial-dependent cellular mechanism as shown in Figure 4 (Scheme 2). According to this theory, the Ca^2+^ and phosphates ions in blood plasma are transported into the cell cytoplasm. Further, the ions are pumped into the inner wall of mitochondria on specific carriers by energy-yielding electron transport (Rossi et al., 1967). When the solubility product of ACP is exceeded, precipitation of micro-packets occurs. The Adenosine triphosphate (ATP) dependent process continues resulting in electron opaque granules composed of many ACP micro-packets which are stabilized by the Howard factor. The micro-packet of ACP can transfer from the mitochondria in two ways; 1) Direct transfer through a mitochondrial membrane or 2) by reverse phagocytosis, leading to out pocketing of membrane vesicles containing two or more micro-packets. After ejecting these micro packets into the extracellular site, they attach to the epitaxial structure of collagen and become part of an amorphous bone mineral fraction. These micro-packets were identified as Posner clusters which possess a neutral ion cluster of 10Å in the longest dimension with a probable chemical structure Ca_9_(PO_4_)_6_ (Betts et al., 1975).

In both, the hypothesis of the role of the Howard factor was crucial in stabilizing ACP. This Howard factor was discovered first, in urine samples (Howard and Thomas, 1968). It was classified as a small organic molecule synthesized by cells, organic in nature, highly acidic, chelating agent and its methylation causes loss of activity. Although there is no evidence about the exact chemical structure of the Howard factor in the literature. Further research was not performed to understand the exact chemical structure of the Howard factor.

### Micro-vesicle theory

In 1967, Bonucci studied the early stage biomineralization in cartilage. The process was initiated by the formation of peri-circular, periodic acid-Schiff positive, osmiophilic, rounded bodies in which the nucleation of apatite nanocrystals was initiated. The crystallites were removed by decalcification leaving “ghost bodies,” which were organic in nature like protein absorbed on the surface of the apatite nanocrystals. The formations of these bodies seem unclear but were believed that these bodies can be formed enzymatically or were secreted by cells. Electron microscopy analysis did not show any structure in these bodies until the initiation of biomineralization. However, these bodies were formed by a homogeneous substance that possesses variable electron density (Bonucci, 1967). In the same year, Anderson also studied the development of cartilage and biomineralization. The electron microscopy studies revealed that the culture of human amniotic cells (FL strain) induces the deposition of a radial cluster-like apatite nanocrystal in the cartilage matrix. This bears a resemblance to the native pattern observed at the epiphysis. Both these studies observed electron-dense “leaf-like” structures with needle-like projections attached to the collagen fibrils in ossifying cartilage matrix, which was then classified as matrix vesicles (MV) (Anderson, 1967).

**FIGURE 5:**
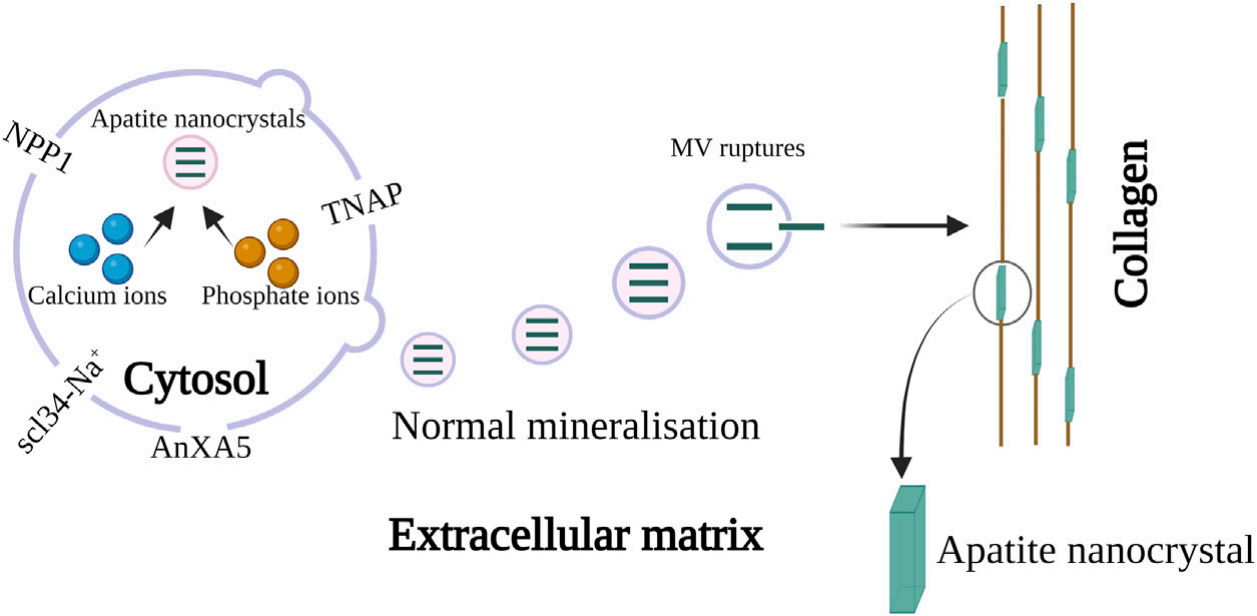
Schematic representation of MV in biomineralization. The biomineralization by MV occurs in two steps, in step 1 apatite nanocrystal is formed *via* the influx of calcium and phosphate ions through AnXA5. The hydrolytic action of the enzyme phospholipase and the protease leads to the penetration of apatite crystal inside MV. Once the MV is released into the extracellular matrix they bind to collagen. This initiates step 2 resulting in biomineralization in presence of collagen. The image is inspired by the artwork of (Bottini et al., 2018) and (Bommanavar et al., 2020).

The properties, function, biogenesis, and biological models of micro-vesicles from chondrocytes and osteoblasts are recently reported in the review presented by Bottini et al. (2018). They proposed that one or more annexin family proteins were responsible for the uptake of Ca^2+^ ion in the MV as shown in Figure 5. They assumed that AnxA5 may act as a carrier protein that will transport Ca^2+^ ions from mitochondria to the inner leaflet of the plasma membrane where it can bind to phosphatidylserine (PS). It was postulated that the lipid rafts in the microvilli membrane form a complex of PS, AnxA5, Ca^2+^, and phosphate which accounts for the formation of the MV nucleation core. Further, it was speculated that the transport of Ca^2+^ and phosphate ions from mitochondria to intracellular vesicles (IV) towards the extracellular matrix may initiate biomineralization. The author also described another hypothesis in which empty MV budding from chondrocytes is released from the microvilli-like cell membrane. After they are released in the extracellular medium, Ca^2+^ and phosphate ions are accumulated following nucleation from apatite nanocrystal. Further, the MVs are ruptured by stress or by the activity of phospholipase and the apatite nanocrystals are released in the extracellular medium which mineralizes on collagen fibers (Bottini et al., 2018). Recently, Beck (2019) has described the role of scl34-Na^+^ phosphate transporter in the biomineralization of bone and tooth. The latest review covers the role of MV mediated mechanism with special reference to the crystal ghost in depth (Bommanavar et al., 2020).

### Phosphoprotein

The phosphoproteins containing serine and threonine amino acids are believed to play a role in interfibrillar collagen mineralization. Organic phosphate and carboxylic side chain groups of serine and threonine account for half of all amino acid residues of phosphoprotein in bone (Spector and Glimcher, 1972). The Ca-binding ability is one of the essential requirements for initiating the nucleation of CaP from solution. The phosphate and the carboxylic side chains of phosphoproteins in dentine have shown remarkable Ca-binding ability (Lee et al., 1977). However, Seyer and Glimcher (1977) have mentioned that Ca-binding with phosphoproteins may decrease interfibrillar collagen mineralization because the electrochemical or stereochemical binding of calcium leads to the unavailability of Ca^2+^ ions to react with the inorganic phosphates. This results in preventing one or more physicochemical steps (nucleation, crystal growth, or crystal multiplication) in apatite deposition. Therefore, to understand the interaction of Ca-bonded phosphoprotein with organic phosphates; experiments and ^31^P NMR analysis were performed.

**FIGURE 6:**
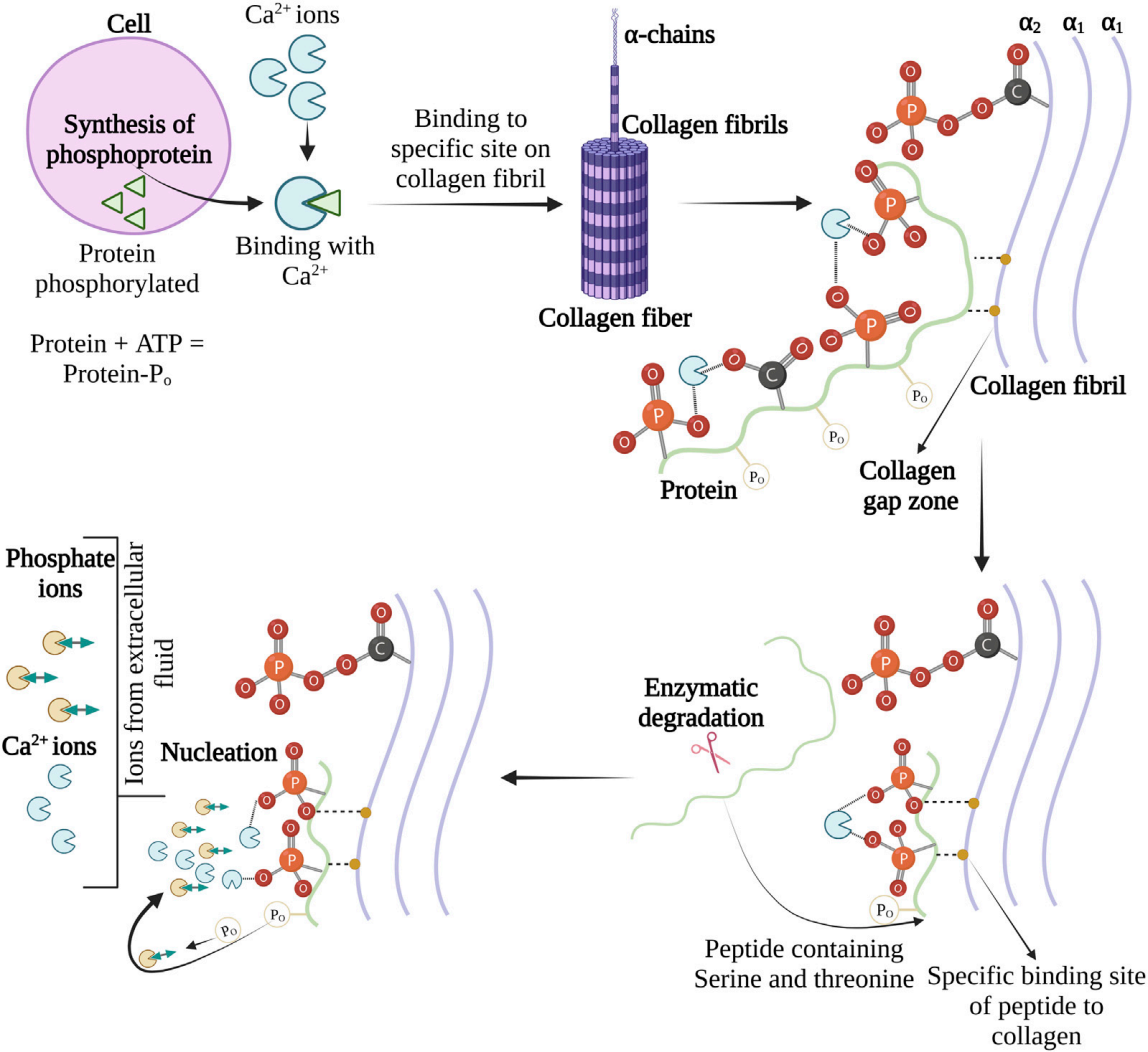
Schema for the role of phosphoproteins interfibrillar collagen mineralization. The image is inspired by the artwork of (Glimcher, 1984).

Initially, the phosphoproteins were titrated with calcium chloride in presence of inorganic orthophosphates, and it was found that in the absence of calcium chloride, there was no shift in ^31^P NMR signals but on the other hand phosphorous peak was observed with broadening or shifting in the presence of calcium chloride. This confirms the formation of a ternary complex between the orthophosphate ions and the proteins phosphomonoester group (Lee et al., 1983). The phosphoprotein-regulated interfibrillar collagen mineralization process is shown in Figure 6. In 1982, Lian and associates have revealed *in vivo* evidence of phosphoproteins in interfibrillar collagen mineralization (Kiebzak et al., 1988).

### Non-collagenous proteins

Collagen account total of 90% organic part of the bone and the remaining part is non-collagenous proteins and low-molecular-weight organic compounds. The non-collagenous proteins are usually referred to as small-integrin binding ligand, N-linked glycoprotein (SIBLING) proteins which consist of osteopontin (OSP), dentin matrix protein (DMP), bone sialoprotein (BSP), dentin sailophosphoprotein (DSPP), and matrix extracellular phosphoglycolproteins (MEPE). These proteins possess the collagen-binding ability as well as RGD sequence for cell binding. Moreover, these proteins share common features of glycosylation, and phosphorylation and have similarities in genomic organization and localization (Huang et al., 2008). A detailed description of the proteins is presented elsewhere (Kalka et al., 2019). Moreover, albumin and α_2_-HS-glycoproteins are present throughout the mineralized bone matrix (McKee et al., 1993). Studies have shown evidence of the involvement of these non-collagenous playing a vital role in interfibrillar collagen mineralization which is explained in the subsequent sections.

### Albumin

Albumin is the major component of blood and one of the important non-collagenous proteins. It possesses a high affinity towards apatite surface inhibiting crystallization. Therefore, it is known as a factor regulation process of interfibrillar mineralization of collagen. Studies on the precipitation and growth mechanism of OCP in collagen, under the influence of serum albumin, revealed the inhibition capacity of albumin in two ways—the association of the carboxyl group of protein with the calcium of OCP and/or adsorption of albumin on the surface of OCP. There was electrostatic interaction between the positively charged OCP and collagen and negatively charged albumin at pH 6.5. The adsorption of albumin at the nucleation site of the collagen surfaces obstructs the nucleation sites of the substrate thus delaying the first nuclei formation. Crystallization of OCP is rapid on the collagen surface, therefore interfibrillar collagen mineralization is hampered. However, the addition of albumin would obstruct the nucleation sites leading to interfibrillar collagen mineralization (Combes et al., 1999).

### α_2_-HS-glycoproteins

In 1944, Fetuin was first isolated from bovine serum and since then most commonly known as Fetuin-A or α_2_-HS-glycoprotein (Mori et al., 2012). α_2_-HS-glycoproteins in the serum inhibit calcium precipitation thus hindering apatite formation. This inhibitory effect was governed by acidic amino acids modulating apatite formation and inhibiting phase separation in serum during the interfibrillar collagen mineralization (Schinke et al., 1996). Further electron microscopy studies have shown that α_2_-HS-glycoproteins with ACP form soluble colloidal spheres. These spheres were termed calciprotein particles that are amorphous in nature with sizes ranging from 30 to 150 nm. The binding of acidic amino acid of α_2_-HS-glycoproteins with ACP inhibits unwanted mineralization outside the interfibrillar collagen (Heiss et al., 2003).

### Oesteopontin and osteocalcin

OCP-osteocalcin complex directs collagen interfibrillar collagen mineralization. Transmission electron microscopy (TEM) analysis revealed that the carboxylate groups in glutamic acid in osteocalcin (OSC) deliver attachment sites to OCP (Pompe et al., 2015). Further, the OSC attaches to collagen and interacts with the Ca-sites of apatite platelets with a Ca-Ca distance of 9.5 Å. Therefore, it was claimed that OSC governs the function of Ca^2+^ ion transport and acts as an intermediary molecule in the nucleation of OCP and apatite in the fibrillar space of collagen (Simon et al., 2018). Further studies revealed the interaction of OSP and OSC with calcium ions induces stability and delays nucleation, whereas interaction with inorganic phosphates results in opposite effects. The study also indicates that OSC concentration negatively influences crystal size and rate of formation though OSC presence results in ordered apatite structures. On the contrary, OSP concentration promotes crystal formation and reduces the Ca:P ratio (Duman et al., 2019).

### Dentin matrix protein

The carboxylic terminal present in the amino acids of the protein plays a crucial role in phosphorylation and binding with Ca^2+^ ions controlling interfibrillar collagen mineralization in dentin (George et al., 1993). Tsuji et al. (2008) synthesized artificial motifs analogous to dentin matrix protein. ACP in presence of these proteins increases the molecular mass and the fractal dimensions without affecting the gyration radii. The protein thus enables the conversion of ACP to apatite. The nucleation initiates at the small radius of the growing crystal till the crystal reaches a critical size (termed a critical radius), following rapid crystal growth. This critical radius can be expressed by the function of temperature, supersaturation, specific internal energies, and volume of growth units.

### Low-molecular-weight organic compounds

#### Parathyroid hormone

The role of parathyroid hormone in calcium metabolism was discovered in 1909 (MacCallum and Voegtlin, 1909). The mechanism of action of the parathyroid hormone was well described in the previous review articles (Rasmussen, 1961; Potts et al., 1982). In 1956, Neuman et al. (1956) postulated a mechanism under the control of the parathyroid gland that was maintaining gradients of calcium ions between blood and bone. The cellular element in the bones secreted citrates in response to parathyroid activity. It was assumed that the citrates carrying calcium complex from serum to skeletal tissue get citrated and oxidized to form several calcium ions. If citrates were secreted in the form of citric acid, then due to the pH gradient ionized calcium ions will be transported to serum. The hypothesis was supported by the *in vitro* enzyme studies. It was found that the enzyme isocitrate dehydrogenase, which is required for citrate utilisation was absent in mature bone. Moreover, the parathyroid hormone destroys the chromophore groups of Coenzyme II. Blocking Coenzyme II-linked reactions shuts glucose metabolism to citrate production. This provides a captivating biochemical mechanism governed by parathyroid hormone in regulating citrate gradient that is responsible for maintaining steady but supersaturated levels of calcium ions in serum.

#### Citrate

Citrates are small organic moieties that originate in the citric acid cycle and play a critical role in the human metabolic pathway. In 1941, the presence of citrate in bone was first identified (Dickens, 1941). Since then, it is known that citrate concentration in hard tissues is in the range of 20–80 μmole/g which is 100–400 folds higher than in most of the soft tissues. It should also be indicated that the bone consists of 1.6% of citrate and about 90% of total body citrates are found in human bone (Granchi et al., 2019). Such a high concentration of citrates in bone indicates their vital role in mineralization. Recent NMR studies have shown the presence of citrate in bone (Davies et al., 2014). The lattice orientation, particle size, and distribution of the apatite are regulated by the complex interaction of the citrate with the apatite. The long axis of the citrate molecule is parallel to the surface of the apatite. The three carboxylic groups of citrates are at 0.3–0.45 nm from the apatite surface. The spacing of the carboxylic groups matches with the calcium ion along the c-axis of the apatite. Therefore, the crystal growth is inhibited in the direction of thickness but continued in the longitudinal direction. This assists in the formation of a plate-like apatite structure analogous to natural bone (Xie and Nancollas, 2010).

**FIGURE 7:**
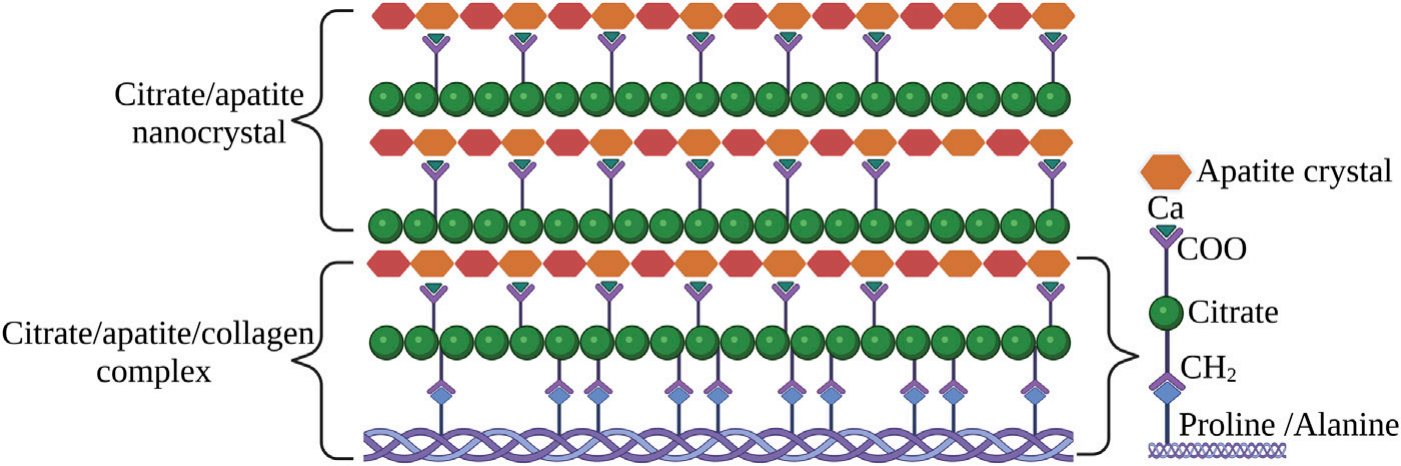
The status of citrate in apatite/collagen component of bone. The image is inspired by the artwork of (Costello et al., 2018).

The occurrence of citrate in bone and its relationship was well described by Costello et al. (2012). The author coined the term “citration” which describes the relationship between citrate-apatite nanocrystals. In further studies, the author also described the potential mechanism of how citrate is involved in interfibrillar collagen mineralization (Costello et al., 2015). The collagen/apatite structure incorporated with citrate in the apatite crystal was depicted as shown in Figure 7.

#### Succinate

Succinic acid (SA) is an intermediate of a citric acid cycle. It can be detected in the bones in exceedingly small concentrations. For instance, 1 g of bone consists of 3.6 mg of SA (Lees and Kuyper, 1957). Like citrates, the carboxylic groups in SA are chemisorbed on the apatite surface by coordinating with calcium ions and regulating the growth and morphology of the crystals. Studies on mineralization reported that SA binds to collagen by hydrogen bonding and encourages interfibrillar collagen mineralization. The interaction of SA with collagen improved the physicochemical properties of apatite and attract more calcium ions, thus leading to accelerated interfibrillar collagen mineralization. Moreover, the SA pre-treated demineralized dentin promoted dentin remineralization. The mechanical properties of the remineralized dentin were similar to natural dentin (Jin et al., 2022). Earlier efforts to synthesize OCP incorporated with SA have been commenced by Marković et al. (1994). They uncovered pH dependency on conversion from SA-OCP to apatite.

#### Amino acids

Apatite formation was observed near the crossband of collagen where basic and acidic amino acids are thought to be more concentrated (Robinson and Watson, 1955). Soon after this finding, Solomons and Irving (1958) studied the relationship of Є-amino acid of lysine and hydroxylysine in mineralization. The Є-amino acid may provide potential phosphate binding sites on the collagen, leading to its phosphorylation. This was assumed to be the first step in matrix interfibrillar collagen mineralization (Weidmann, 1963). Ikawa et al. (2009) have synthesized amino acid-containing ACP and investigated its transformation in a simulated body fluid. They found that the incorporation of amino acids enhanced the solubility of ACP and lead to faster conversion to apatite. The role of amino acids in mineralization is covered in-depth in a recent review by Tavafoghi and Cerruti (2016).

#### Pyrophosphate

Fleisch and Sylvia demonstrated *in vitro* nucleation and mineralization of apatite nanocrystals in presence of collagen (Herbert and Bisaz, 1962). They have proposed the role of pyrophosphate in regulating biomineralization. In the nucleating region of collagen, the precipitation of apatite nanocrystals occurs at the physiological concentration of the Ca^2+^ and phosphate ions. It was depicted that the inorganic pyrophosphates (PP_i_) present in blood plasma protect the nucleation site in the collagen inhibiting growth of apatite nanocrystals. For the mineralization of collagen, the pyrophosphates must be inactivated by the enzyme pyrophosphatase which was found in mineralized tissue (Herbert And Bisaz, 1962). Further studies conducted by Moochhala et al. (2008) explored the role of proteins and genes in controlling biomineralization. The enzyme nucleoside pyrophosphatase phosphodiesterase (NPP1) acts on nucleoside triphosphate to generate extracellular PP_i_. Harmey et al. (2004) have further demonstrated hyper-mineralization in *enpp1* (NPP1) knockout mice, resulting in peripheral joint hyperostosis intervertebral fusion. Moreover, the role of the *ank* gene (ANKH in humans) in exporting PP_i_ from cytoplasm to extracellular space was noticed. The *ank* protein was linked with tissue calcification in humans and mice. The third gene *akp2* encodes tissue non-specific alkaline phosphatase (TNAP) which hydrolyses PP_i_, and polyphosphates to release inorganic phosphate. The TNAP activity promotes biomineralization by lowering PP_i_ levels and increasing levels of inorganic phosphate.

#### Polymer-induced liquid precursors

Polymer-induced liquid precursors (PILP) are amorphous mineral precursors stabilized by charged polymers. In the early 1990s, the role of acidic macromolecules in the biomineralization of bone and seashells was known. The acidic macromolecule was known to control the morphology of the inorganic crystal. However, the function of the acidic macromolecule was not clarified. In 1998, Gower and Tirrell (1998) have discovered that the addition of a small amount of polyaspartic acid (pAap) results in CaCO_3_ helices and films. The polymer acts as a membrane and supports the deposition and growth of CaCO_3_. The charge polymer ion inhibits crystal nucleation by inducing liquid-liquid phase separation in the crystallizing fluid. Droplets from the fluidic amorphous phase accumulate and combine resulting in mineral films. Olszta et al. (2007) have postulated the role of PILP biomineralization of bone and teeth. They suggest that the apatite nanocrystal does not nucleate within the gap zone, rather a liquid amorphous phase is pulled into collagen fibers by capillary actions. Once the liquid precursor phase is converted to the solid amorphous phase, crystallization occurs resulting in mineralized collagen with apatite nanocrystals. A combination of polyanionic non-collagenous proteins was presumed to be analogous to PILP. This hypothesis was backed by cryo-TEM analysis by Nudelman et al. (2010). They discovered that the electrostatic interaction between pAap-ACP complex and the cationic amino acids in the gap zone of collagen assists infiltration of PILP. Citrate also plays a crucial role in PILP by enhancing the degree of collagen mineralization *in vitro*. The citrate reduces the interfacial energy between collagen and PILP leading to an increase in wettability (Shao et al., 2018). Polyacrylic acid (PAA) was also used successfully in PILP for the stabilization of ACP. PAA of various molecular weights was analyzed, and it was found that both low and high-concentration PAA stabilized ACP and allows infiltration in the collagen matrix. The unstable ACP results in extra fibrillar mineralization. On the contrary, extensively stabilized ACP did not initiate collagen mineralization. This suggests that the concentration of PAA plays a crucial role in regulating interfibrillar collagen mineralization (Shen et al., 2021).

## Discussion

It is known that collagen plays a crucial role in the biomineralization process. The unique physiochemical properties of collagen offer this potential. Based on the physical characteristics of hydration a model of collagen interfibrillar biomineralization was demonstrated. Molecular dynamic studies have shown the balance between osmotic equilibrium and electroneutrality establishes Gibbs-Donnan equilibrium in a polyelectrolyte-directed system (Niu et al., 2017). Based on the chemical properties of collagen numerous models are presented. For instance, the amino acid residue in the two-dimension structure of type-I collagen molecular segment as well as in the adjacent segments of the triple helix chain. The charge distribution of the peptide in the collagen chain offers charged domain that serves as a center for apatite formation. However experimental studies have shown that the synthetic ACP/collagen combination alone is not capable of initiating the nucleation of apatite rapidly (Bradt et al., 1999). This indicates that collagen cannot initiate or regulate interfibrillar mineralization alone (Posner, 1969). Therefore, the role of non-collagenous proteins and other ECM molecules was considered to affect mineralization either by enhancing collagen mineral interaction or binding mineral ions. However, there are numerous postulations on this phenomenon, but the exact mechanism is poorly understood (Silver and Landis, 2011).

There are numerous mechanisms for how calcium phosphate is deposited on the collagen matrix. Different organic compounds are believed to regulate interfibrillar mineralization. Most of these compounds possess a carboxyl functional group. The carboxyl group is a combination of two functional groups in which a single carbon atom is attached to hydroxyl (−OH) and carbonate (=O) groups. This makes the carbonyl group polar, highly electronegative, and weakly acidic, capable of hydrogen bonding by accepting or donating proton (Klecker and Nair, 2017). The carboxylate anion has the capability of forming bonds with calcium by direct binding or by influencing electrostatic interactions away from the metal center (Chakrabarti, 1990). Moreover, the carboxylate ions also have the potential to react with phosphate *via* the formation of P-O-H-O-C bonding (Corbridge, 1971). Owing to these exclusive properties, carboxylate-incorporated calcium phosphate materials are gaining more interest.

In the past 20 years, the research is focused on finding the role of carboxylic compounds in interfibrillar collagen mineralization (Ikawa et al., 2008; Sakamoto et al., 2008; Ishikawa and Oaki, 2014; Josipović et al., 2020; Veiga et al., 2020; Wang et al., 2020). Recent studies have also provided new findings such as A) Influence of carboxylic ions in the conversion of ACP to OCP (Sugiura et al., 2015). B) The density of carboxylic ions plays a crucial role in controlling the growth and mineralization of calcium phosphates (Zhang et al., 2022). C) In the early nucleation, citrate binds to ACP and controls the size and morphology of apatite crystal (Xie and Nancollas, 2010). D) One-sixth of the available apatite surface area in bone is covered by citrate (Xie and Nancollas, 2010). E) Carboxylate ions can only be incorporated into the hydrated layer of CaP (Yokoi et al., 2022). F) Collagen matrix is denser and irregularly distributed in the bone as compared to the majority of other soft tissues (Tzaphlidou, 2008).

## Conclusion

Interfibrillar collagen mineralization has gained attention in the past 20 years, the major reason is the abundance of both apatite nanocrystals and collagen in bone. Studies have indicated the role of physiochemical properties of collagen as well as non-collagenous proteins and low molecular weight compounds regulating the process. New insights such as the role of genes and enzymes in this process are also discovered. Overall, the process of interfibrillar collagen mineralization is complex. However, advanced analysis techniques such as cryo-TEM, Cryo-scanning electron microscopy, X-Ray tomography, molecular dynamics, gene knockout, metabolomics, proteomics, DNA and RNA sequencing, etc. allow investigation of mechanism within nanometer resolution while maintaining the structure close to the native structure. These advanced analysis techniques with a combination of both *in vivo* and *in vitro* analysis potentially answer all the underlying questions in the future. However, the process is currently in the preliminary stage, but the technological advancement will soon decode the process of interfibrillar collagen mineralization.
